# Supplementary material for: Determinate growth is predominant and likely ancestral in squamate reptiles
Source: Proc Biol Sci. 2020 Dec 23;287(1941):20202737. doi: 10.1098/rspb.2020.2737 (PMC7779497; doi:10.1098/rspb.2020.2737)
Supplement: Supplementary Material [file rspb20202737supp1.pdf]

Electronic Supplementary Material

Determinate growth is predominant and likely ancestral in squamate reptiles

Petra Frýdlová, Jana Mrzílková, Martin Šeremeta, Jan Křemen, Jan Dudák, Jan Žemlička, Bernd Minnich, Kristina Kverková, Pavel Němec, Petr Zach, Daniel Frynta

Corresponding authors: Petra Frýdlová, petra.frydlova@seznam.cz, **Daniel Frynta,** frynta@centrum.cz

Department of Zoology, Faculty of Science, Charles University, Vinicna 7, 128 43 Prague, Czech Republic

Content:

1. Supplementary Methods

Sample size; μRTG and μCT examinations; ancestral state reconstruction; histological and microscopic methods of femoral mid-diaphyseal cross-sections are described.
Table S1. Specification of BiSSE and HiSSE models used for ancestral state analysis

2. Supplementary Results

Outcomes of parsimony and likelihood models are summarized, and data gathered in this study are provided.
Figure S1. Ancestral state reconstruction of growth type in squamates, maximum parsimony model
Figure S2. Ancestral state reconstruction of growth type in squamates, AIC-weighted average of two best fit maximum likelihood models
Figure S3. Ancestral state reconstruction of growth type in squamates, AIC-weighted average of all five acceptable maximum likelihood models
Figure S4. Mid-diaphyseal transverse cross-sections of the femur in four species of adult lizards with resorbed GPC.
Table S2. Epiphyseal state in the proximal epiphysis of the femur in the examined specimens representing clades Gekkota, Scincoidea and Lacertoidea
Video File S1. The visualization of transversal cross-sections of the proximal part of the femur by μCT in adult *Timon tangitanus* (ID 120).
Video File S2. The visualization of longitudinal sections of the proximal part of the femur by μCT in adult *Timon tangitanus* (ID 120).
Video File S3. The visualization of transversal cross-sections of the proximal part of the femur by μCT in adult *Timon tangitanus* (ID 102).
Video File S4. The visualization of longitudinal sections of the proximal part of the femur by μCT in adult *Timon tangitanus* (ID 102). For other visualization of proximal part of the femur from Figure 1 [*Correlophus ciliatus* (ID 314 and 373) and *Zonosaurus karsteni* (ID 299 and 418)] in full resolution see dataset on Dryad (https://doi.org/10.5061/dryad.dbrv15dxz).

3. Supplementary Discussion

Technical considerations

4. Supplementary References

1. Supplementary Methods

Animals

We analysed 194 femoral bones from lizards belonging to three clades of Squamata (Gekkota, Scincoidea, and Lacertoidea), covering much of the species diversity, except for the legless groups. Specifically, we analysed 38/100 species/individuals from Gekkota (1/2 Carphodactylidae, 4/10 Diplodactylidae, 7/18 Eublepharidae, 4/13 Sphaerodactylidae, 3/4 Phyllodactylidae, 19/53 Gekkonidae), 26/53 Scincoidea (7/20 Gerrhosauridae, 2/9 Cordylidae, 17/24 Scincidae) and 21/41 Lacertoidea (7/7 Teiidae, 14/34 Lacertidae).

μRTG and μCT examinations

The Bruker SkyScan 1275 μCT scanner was used for scanning of large samples, while a custom-built μCT system utilizing large-area photon counting detectors based on Timepix technology [1] was used for smaller samples, since higher resolution and higher contrast-to-noise ratio could be achieved using this set-up [2]. The scan parameters were adjusted for each sample individually to reflect its size and attenuation properties (for more technical details see [3, 4]. The voxel-size of the reconstructed slices was within the range of 4 – 13 μm. Data analysis was carried out using Bruker CTVox [5] and Fiji [6].

Ancestral state reconstruction

We fit a total of 12 models of trait evolution and diversification using the R packages hisse [7], including the full HiSSE model, subsets of the full HiSSE model with different constraints on transition rates, BiSSE-like models without hidden states and null-models with diversification rates independent of the observed states (all input data are in Dataset 2).

BiSSE-like model refers to a model in which there are no hidden states, turnover and extinction rates vary across states and there are two transition rates (i.e., state 0 to 1 and 1 to 0). Full HiSSE model refers to a HiSSE model in which each observed state is associated with two hidden states (i.e., states 0A, 0B, 1A, 1B), turnover and extinction rates vary across all four states, and there are eight character transition rates, with dual transitions (e.g., state 0A to 1B) not allowed. All other models are subsets of the full HiSSE model with various constraints on diversification rates and transition rates, or “null” models with diversification rates independent of the states but still allowing for varying diversification rates (for hidden states). All models are described in detail in Table S1 below.

In all analyses, we accounted for incomplete species sampling by setting the sampling fraction of species in each state of the observed trait. We estimated the total number of recent species with a given state for each high-level clade, multiplying the number of species in the clade included in the Reptile database [8] by the fraction of species with the given state in our dataset, assuming that our sampling gives a good approximation of the states proportion. For clades not represented in our data, we assumed a conservative proportion of 50% for each state. For the phylogenetic relationships we used a time-calibrated phylogeny of 4162 squamate species [9]. The best model fit was selected based on the Akaike information criterion (AIC) and the composite models were created with AIC weighted average of the model fits with Δ AIC < 4 (2 best models) or 10 Δ AIC < 10 (all acceptable models).

Histological examinations

We analysed histology of femoral bones in four fully grown individuals representing phylogenetically distantly related species, namely at least 9-year old female of Yellow-throated plated lizard *Gerrhosaurus flavigularis* (ID 99); at least 5-year old male of Balkan green lizard *Lacerta trilineata major* (ID 640); nearly 12-year old male of Kuhl's flying gecko *Ptychozoon kuhli* (ID 358) and at least 7-year old female of Common leopard gecko *Eublepharis macularius* (ID 187). All these individuals

were captive bred and featured complete resorption of the GPC. Samples were fixed in ethanol, decalcified in 8% nitric and 8% hydrochloric acid solution for 7 hours, dehydrated in graded ethanol series and embedded in paraffin [10]. Mid-diaphyseal regions of femoral bones were transversally sectioned at a 15μm thickness by a rotary microtome. Diaphyseal cross-sections were mounted on glass slides, stained with Ehrlich's haematoxylin and examined under bright field illumination, phase contrast, Nomarski interference contrast and in polarized light at 400x magnification. A minimum of thirty sections per bone were examined.

Table S1. Specification of BiSSE and HiSSE models used for ancestral state analysis

Model	Hidden states	State-dependent diversification	Diversification rates	Transition rates	AIC
**BiSSE-like**	No	Yes	2	2	1853
**BiSSE-like equal diversification**	No	No	1	2	1847
**HiSSE full**	Yes	Yes	4	8	1853
**HiSSE equal rate**	Yes	Yes	4	1	1831
**HiSEE equal hidden equal rate**	Yes	Yes	3	1	1836
**HiSSE equal hidden eight rates**	Yes	Yes	3	8	1834
**HiSSE equal diversification**	Yes	No	1	8	1851
**HiSSE null-four three-rates**	Yes	No	4	3	1839
**HiSSE null-four equal rate**	Yes	No	4	1	1836
**HiSSE null-three eight rates**	Yes	No	3	8	1847
**HiSSE null-two eight rates**	Yes	No	2	8	1848
**HiSSE null-two equal rate**	Yes	No	2	1	1841

2. Supplementary Results

Ancestral state reconstruction

Maximum parsimony model suggests that the last common ancestors of squamate reptiles was a determinate grower (Figure S1), as do the maximum likelihood models (Figures 2, S2, S3). The best-fit likelihood model (the HiSSE equal rate model with state-dependent diversification rates and transition rates equal across observed and hidden states) support determinate growth as the ancestral state for Squamata (85.5%, Figure 2), the same is true for model-averaged reconstructed states for the two best models (ΔAIC < 3; 88%, Figure S2) and all acceptable models (ΔAIC < 10; 85%, Figure S3).

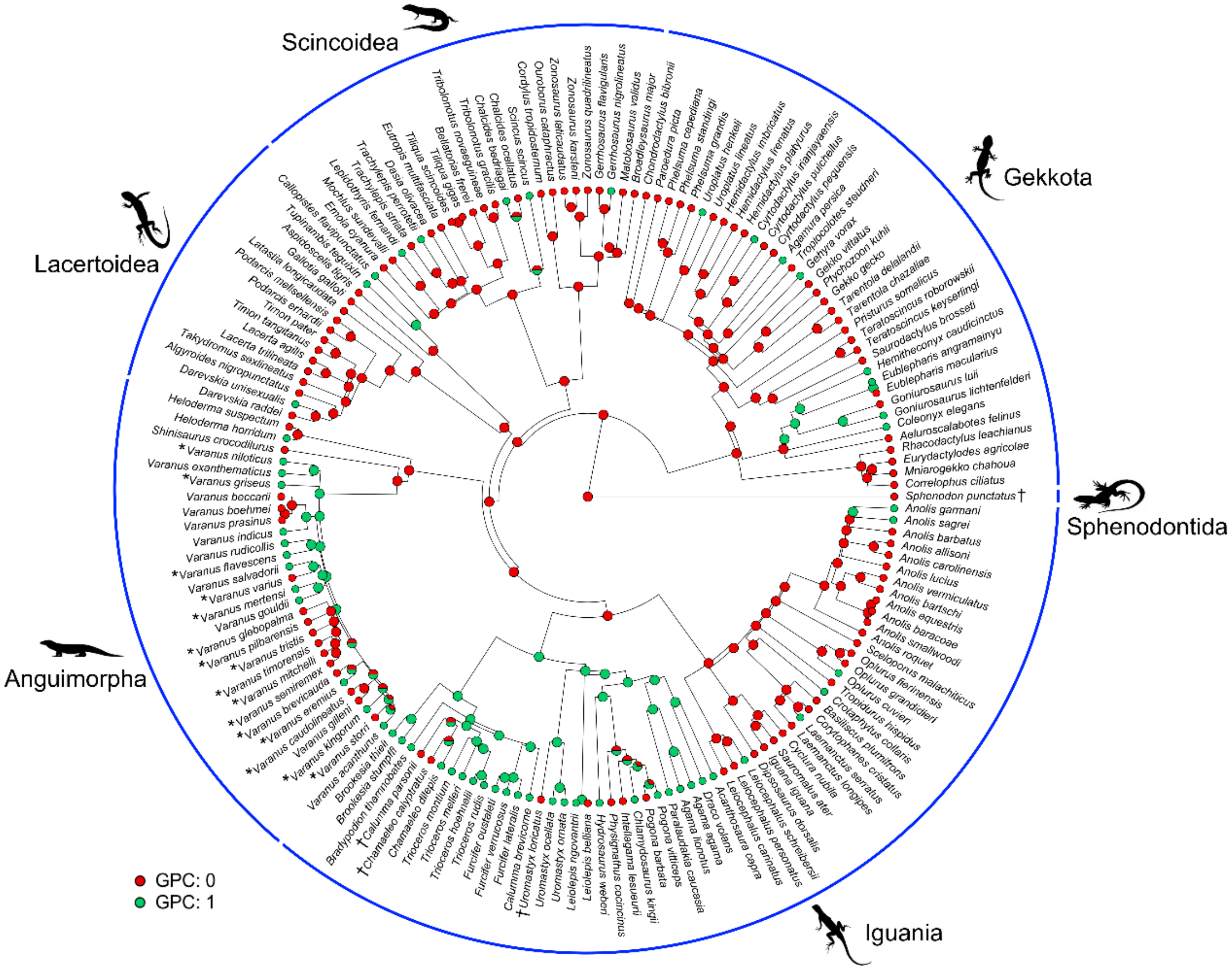

Figure S1. Ancestral state reconstruction of growth type in squamates, maximum parsimony model. A circular tree depicting the growth plate cartilage (GPC) state in whole Squamata as revealed by µRTG and µCT examination of the proximal part of femoral bones. Ancestral state reconstruction method was employed using parsimony model to uncover the evolution of growth type (determinate vs. indeterminate) in Squamata. GPC present (green) and absent (red) is suggesting extended (potentially indeterminate) vs determinate body growth. Tuatara (*Sphenodon punctatus*), as a sister group of Squamata, was included as an outgroup. The state of tuatara is according to the presence of external fundamental system and recapture growth data suggesting the determinate type of body growth [11, 12]. Species marked with asterisk were scored according to the GPC state from literature [13]. Species marked with † were very old individuals (for details of age see references [3] and [4]).

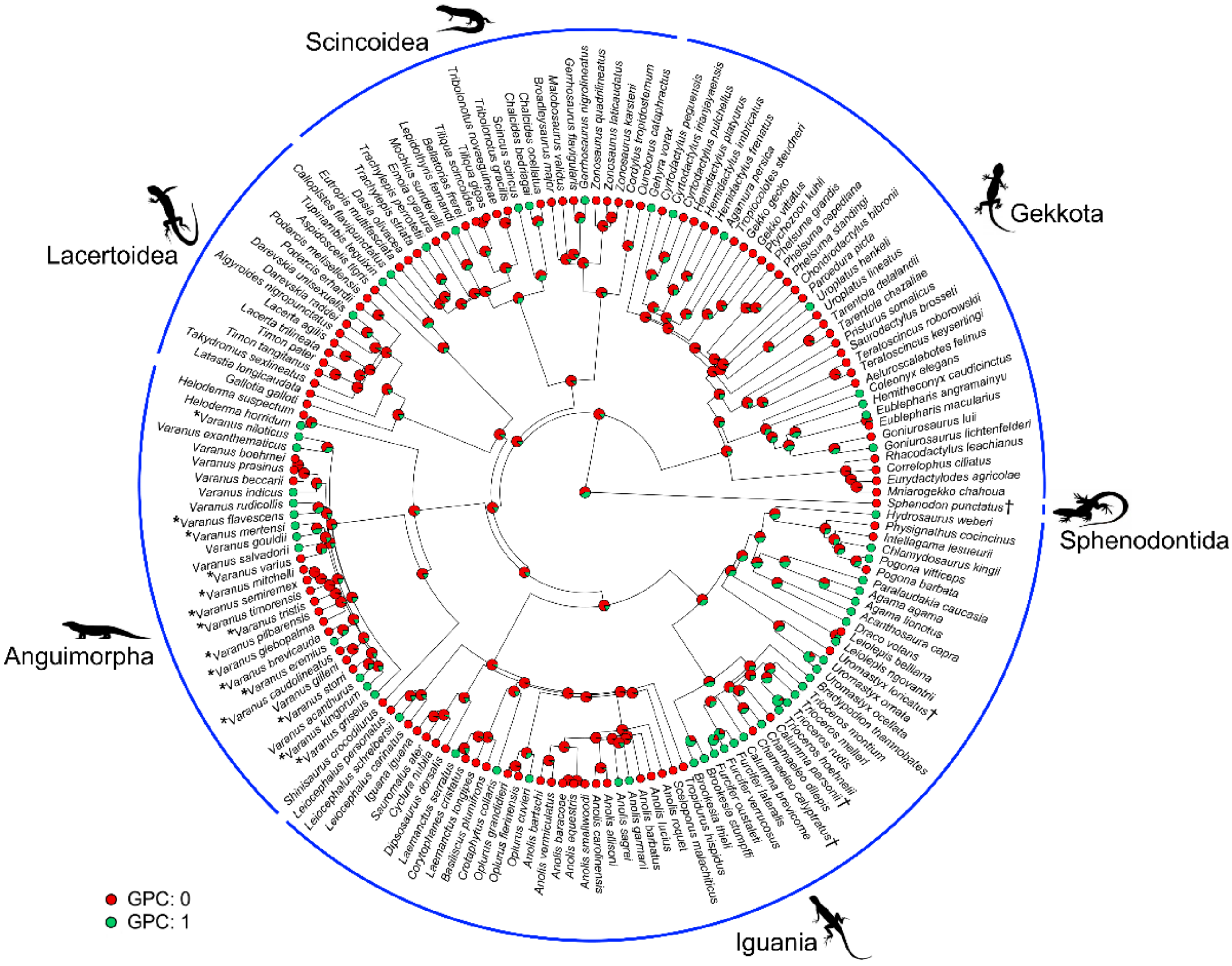

Figure S2. Ancestral state reconstruction of growth type in squamates, AIC-weighted average of two best fit maximum likelihood models. A circular tree depicting the growth plate cartilage (GPC) state in whole Squamata as revealed by μRTG and μCT examination of the proximal part of femoral bones. Ancestral state reconstruction method was employed using maximum likelihood with hidden state speciation and extinction models to uncover the evolution of growth type (determinate vs. indeterminate) in Squamata. GPC present (green) and absent (red) is suggesting extended (potentially indeterminate) vs determinate body growth. Tuatara (*Sphenodon punctatus*), as a sister group of Squamata, was included as an outgroup. The state of tuatara is according to the presence of external fundamental system and recapture growth data suggesting the determinate type of body growth [11, 12]. Species marked with asterisk were scored according to the GPC state from literature [13]. Species marked with † were very old individuals (for details of age see references [3] and [4]).

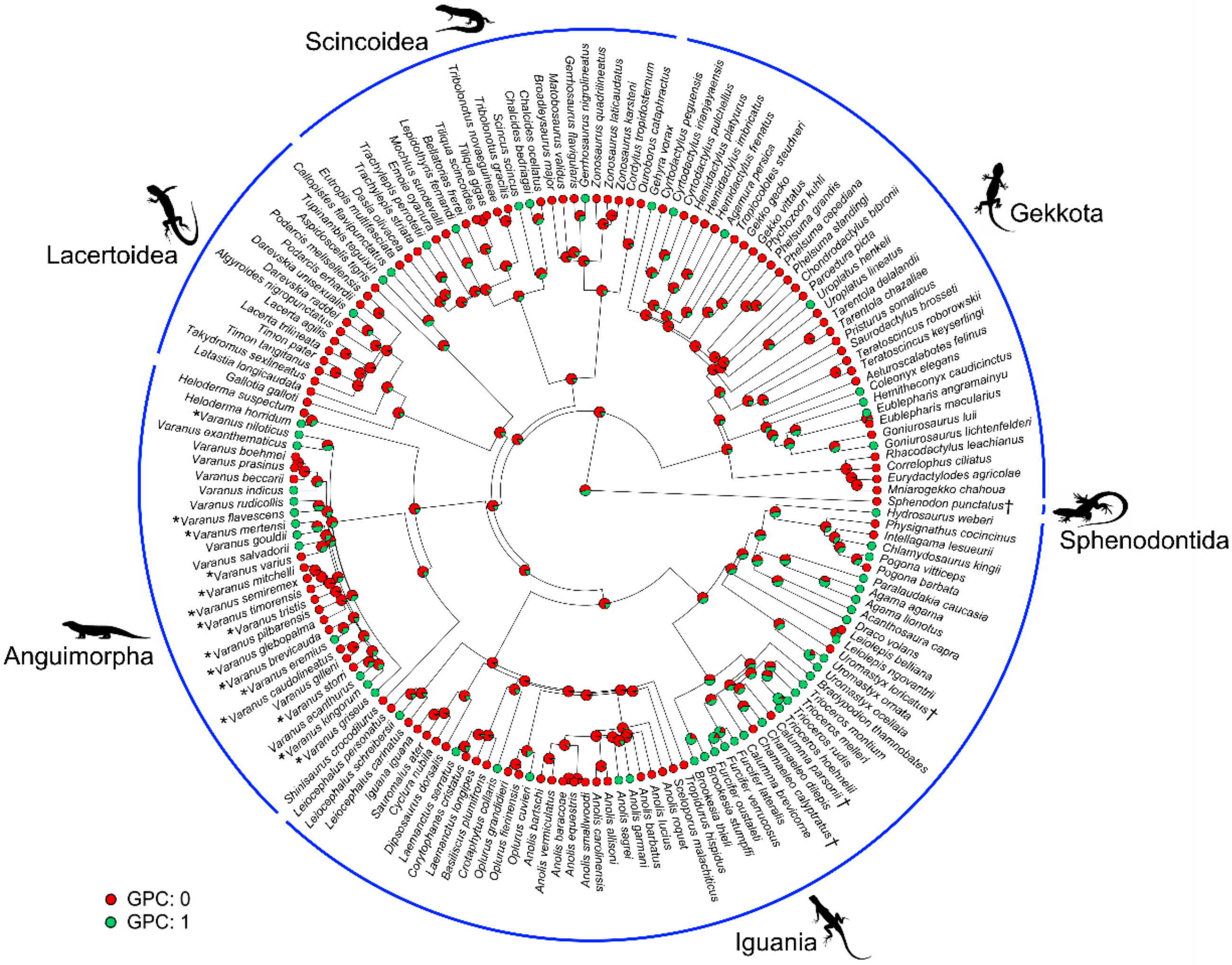

Figure S3. Ancestral state reconstruction of growth type in squamates, AIC-weighted average of all five acceptable maximum likelihood models. A circular tree depicting the growth plate cartilage (GPC) state in whole Squamata as revealed by µRTG and µCT examination of the proximal part of femoral bones. Ancestral state reconstruction method was employed using maximum likelihood with hidden state speciation and extinction models to uncover the evolution of growth type (determinate vs. indeterminate) in Squamata. GPC present (green) and absent (red) is suggesting extended (potentially indeterminate) vs determinate body growth. Tuatara (*Sphenodon punctatus*), as a sister group of Squamata, was included as an outgroup. The state of tuatara is according to the presence of external fundamental system and recapture growth data suggesting the determinate type of body growth [11, 12]. Species marked with asterisk were scored according to the GPC state from literature [13]. Species marked with † were very old individuals (for details of age see references [3] and [4]).

μRTG and μCT examinations

Video File S1. The visualization of transversal cross-sections of the proximal part of the femur by μCT in adult *Timon tangitanus* (ID 120). The growth plate cartilage is present. Bar = 1000 μm. For full resolution see dataset on Dryad.

Video File S2. The visualization of longitudinal sections of the proximal part of the femur by μCT in adult *Timon tangitanus* (ID 120). The separation of proximal epiphysis from the metaphyseal region and diaphysis of the femur by a radio-translucent layer of the non-calcified growth plate is apparent. The suture between epiphysis and metaphysis is present. Bar = 1000 μm. For full resolution see dataset on Dryad.

Video File S3. The visualization of transversal cross-sections of the proximal part of the femur by μCT in adult *Timon tangitanus* (ID 102). The growth plate cartilage is completely resorbed. Bar = 1000 μm. For full resolution see dataset on Dryad.

Video File S4. The visualization of longitudinal sections of the proximal part of the femur by μCT in adult *Timon tangitanus* (ID 102). The epiphyseal growth plate is absent. The suture between epiphysis and metaphysis is not present and the endosteal bone trabeculae are expanded into the metaphysis. Bar = 1000 μm. For full resolution see dataset on Dryad.

Table S2. Epiphyseal state in the proximal epiphysis of the femur in the examined specimens representing clades Gekkota, Scincoidea and Lacertoidea.

Growth plate cartilage (GPC) presence (1), absence (0), and under the process of degradation (*), Snout-Vent Length (SVL) in millimetres, SVLrel is relative SVL (in % of maximal SVL from the literature), Sex and Age in years, where known. Abbreviations: Male (M), Female (F), Adult (A), Subadult (SA), Individual identity (ID), Charles University (CUNI), National Museum (NMP), Zoo (Z), Wild origin(W), Captive origin (C).

Clade	Family	Species	GPC	SVL	SVL_{rel}	Sex	Age	ID	Source	W/C
Gekkota	Carphodactylidae	*Nephrurus levis*	1	77	78.33	F	A	537	CUNI	C
		Nephrurus levis	1	76	77.31	F	A	597	CUNI	C
	Diplodactylidae	*Correlophus ciliatus*	1	95	73.08	F	A	167	CUNI	C
		Correlophus ciliatus	0	109	83.85	F	A	176	CUNI	C
		Correlophus ciliatus	1	102.73	79.02	M	A	314	CUNI	C
		Correlophus ciliatus	0	97	74.62	M	A (>11.5 y)	373	CUNI	C
		Eurydactylodes agricolae	0	67	128.85	F	A (>5 y)	376	CUNI	C
		Eurydactylodes agricolae	0	49	104.26	M	A (5 y)	377	CUNI	C
		Eurydactylodes agricolae	1	48	102.13	M	A	602	CUNI	C
		Mniarogekko chahoua	0	122	82.99	M	A	63	Dubeč Z.	C
		Rhacodactylus leachianus	0	197	80.41	F	A	62	Dubeč Z.	C
		Rhacodactylus leachianus	0	170	69.39	M	A	259	Dubeč Z.	C
	Eublepharidae	*Aeluroscalabotes felinus*	0	100	87.05	F	A (3 y)	249	CUNI	C
		Aeluroscalabotes felinus	0	98	106.92	M	A (10 y)	374	CUNI	C
		Aeluroscalabotes felinus	0	76	66.16	F	A	375	CUNI	C
		Coleonyx elegans	1	88	96.49	F	A	599	CUNI	C
		Eublepharis angramainyu	1	144	92.07	F	A	181	CUNI	C
		Eublepharis macularius	0	110	85.47	F	A (7 y)	186	CUNI	C
		Eublepharis macularius	0	102	79.25	F	A (7 y)	187	CUNI	C
		Eublepharis macularius	0	120	93.24	F	A	652	CUNI	C
		Eublepharis macularius	0	118	91.69	F	A	657	CUNI	C
		Eublepharis macularius	0	125	97.13	F	A (>7 y)	121	CUNI	C
		Eublepharis macularius	0	119	92.46	F	A (>5 y)	122	CUNI	C
		Eublepharis macularius	0	129	93.61	M	A (>4 y)	123	CUNI	C
		Eublepharis macularius	0	125	97.13	F	A (>7 y)	124	CUNI	C
		Goniurosaurus lichtenfelderi	1	90	90.68	F	A	67	Dubeč Z.	C
		Goniurosaurus luii	0	108	92.19	M	A (3 y)	243	CUNI	C
		Hemitheconyx caudicinctus	1*	93	72.43	F	A	64	Dubeč Z.	C
		Hemitheconyx caudicinctus	1	113	88.01	F	A	65	Dubeč Z.	C
		Hemitheconyx caudicinctus	1	105	72.66	M	A	362	Zájezd Z.	C
	Gekkonidae	*Agamura persica*	1	65	86.91	F	A	604	CUNI	W

Clade	Family	Species	GPC	SVL	SVL_{rel}	Sex	Age	ID	Source	W/C
		Agamura persica	1	60	80.22	M	A	605	CUNI	W
		Agamura persica	1	62	82.90	-	A	606	CUNI	W
		Cyrtodactylus irianjayaensis	1	158.9	97.48	M	A	208	CUNI	W
		Cyrtodactylus peguensis	0	79	92.94	F	A (3.5 y)	250	CUNI	C
		Cyrtodactylus pulchellus	0	97	84.35	M	A	600	CUNI	W
		Gehyra vorax	1	112	71.79	M	A	183	CUNI	W
		Gehyra vorax	1	128.5	91.13	F	A	197	CUNI	W
		Gekko gecko	1	131.6	86.58	F	A	301	CUNI	W
		Gekko gecko	0	162.12	95.36	M	A	306	CUNI	W
		Gekko gecko	1*	140	82.35	M	A	587	CUNI	C
		Gekko vittatus	0	101.9	99.71	M	A	209	CUNI	W
		Gekko vittatus	0	90	88.06	F	A	635	CUNI	C
		Gekko vittatus	1	97	94.91	M	A	636	CUNI	C
		Hemidactylus frenatus	0	60.4	123.01	F	A	211	CUNI	W
		Hemidactylus imbricatus	0	50	96.15	M	A	180	CUNI	C
		Hemidactylus imbricatus	0	51	98.08	M	A	598	CUNI	W
		Hemidactylus imbricatus	1	52	100.00	F	A	607	CUNI	W
		Hemidactylus imbricatus	1	51	98.08	F	A	608	CUNI	W
		Hemidactylus imbricatus	1	45	86.54	F	A	609	CUNI	W
		Hemidactylus platyurus	1	57	98.11	M	A	637	CUNI	W
		Hemidactylus platyurus	0	55	108.48	F	A	639	CUNI	W
		Chondrodactylus bibronii	0	94.38	95.82	M	A	198	CUNI	W
		Paroedura picta	1	86.1	81.41	M	A (1 y)	281	CUNI	C
		Paroedura picta	1	77.27	83.09	F	A (1 y)	282	CUNI	C
		Paroedura picta	1	74.45	80.05	F	A (1 y)	283	CUNI	C
		Paroedura picta	1	84.72	80.11	M	A (1 y)	284	CUNI	C
		Paroedura picta	1	82.12	88.30	F	A (1 y)	285	CUNI	C
		Paroedura picta	1	84.79	80.17	M	A (1 y)	286	CUNI	C
		Paroedura picta	1	81.3	76.87	M	A (1 y)	287	CUNI	C
		Paroedura picta	1	78.28	84.17	F	A (1 y)	288	CUNI	C
		Paroedura picta	1	88.38	83.57	M	A (1 y)	289	CUNI	C
		Paroedura picta	1	84.65	91.02	F	A (1 y)	290	CUNI	C
		Paroedura picta	1	96.9	91.62	M	A (1 y)	311	CUNI	C
		Paroedura picta	0	104.39	98.70	M	A (4 y)	182	CUNI	C
		Phelsuma cepediana	0	52	89.66	M	A	15	CUNI	C
		Phelsuma cepediana	0	43	74.14	M	A	42	CUNI	C
		Phelsuma grandis	0	97.4	95.12	M	A	298	CUNI	C

Clade	Family	Species	GPC	SVL	SVL$_{rel}$	Sex	Age	ID	Source	W/C
		Phelsuma standingi	0	113.6	84.15	M	A	297	CUNI	C
		Phelsuma standingi	1	60	44.44	F	SA	384	CUNI	C
		Phelsuma standingi	0	100	74.07	F	A	451	CUNI	C
		Phelsuma standingi	1	78	57.78	F	A	452	CUNI	C
		Ptychozoon kuhli	0	85	78.85	M	A (>11.5 y)	358	CUNI	C
		Ptychozoon kuhli	0	84	77.92	F	A	588	CUNI	C
		Ptychozoon kuhli	0	86	79.78	M	A	594	CUNI	C
		Tropiocolotes steudneri	0	30.79	96.22	M	A	437	CUNI	W
		Uroplatus henkeli	1	150	93.75	M	A	31	CUNI	C
		Uroplatus henkeli	1	140	87.50	M	A	32	CUNI	C
		Uroplatus henkeli	1	140	87.50	F	A	33	CUNI	C
		Uroplatus henkeli	1	135	84.38	F	A	34	CUNI	C
		Uroplatus henkeli	1	128	80.00	F	A	41	CUNI	C
		Uroplatus lineatus	1	118	90.56	M	A	39	CUNI	C
		Uroplatus lineatus	0	125	95.93	M	A	40	CUNI	C
	Phyllodactylidae	*Tarentola annularis*	1	90	64.29	M	A	630	CUNI	W
		Tarentola delalandii	0	58.92	101.59	M	A	202	CUNI	W
		Tarentola chazaliae	0	58	101.75	F	A	235	CUNI	C
		Tarentola chazaliae	0	64	112.28	F	A (7 y)	356	CUNI	C
	Sphaerodactylidae	*Pristurus somalicus*	0	35	89.74	M	A	388	CUNI	W
		Pristurus somalicus	0	34	87.18	M	A	389	CUNI	W
		Saurodactylus brosseti	0	29	93.55	M	A (4 y)	248	CUNI	C
		Teratoscincus keyserlingii	0	113	103.27	F	A (>14 y)	240	CUNI	C
		Teratoscincus keyserlingii	1	102	93.22	F	A	612	CUNI	C
		Teratoscincus keyserlingii	1	101	92.30	F	A	613	CUNI	C
		Teratoscincus keyserlingii	1	105	95.96	F	A	614	CUNI	C
		Teratoscincus keyserlingii	1	93	84.99	F	A	615	CUNI	C
		Teratoscincus keyserlingii	1	95	86.82	F	A	616	CUNI	C
		Teratoscincus keyserlingii	1	85	77.68	F	SA	623	CUNI	C
		Teratoscincus keyserlingii	0	98	89.56	F	A	624	CUNI	C
		Teratoscincus roborowskii	0	94	123.68	M	A	60	Dubeč Z.	C
		Teratoscincus roborowskii	1	82	107.89	M	A	621	CUNI	C
Lacertoidea	Lacertidae	*Algyroides nigropunctatus*	0	63.76	91.09	M	A	315	CUNI	W
		Darevskia raddei	1	56	87.68	M	A	645	CUNI	W
		Darevskia raddei	0	60	93.94	M	A	646	CUNI	W
		Darevskia unisexualis	1	60	85.71	F	A	642	CUNI	W
		Darevskia unisexualis	1	60	85.71	F	A	643	CUNI	W

Clade	Family	Species	GPC	SVL	SVL_{rel}	Sex	Age	ID	Source	W/C
		Darevskia unisexualis	1	52	74.29	F	A	644	CUNI	W
		Gallotia galloti	0	111.2	91.15	M	A	312	CUNI	C
		Gallotia galloti	0	112	109.80	F	A	618	CUNI	C
		Gallotia galloti	0	105	87.50	M	A	619	CUNI	C
		Gallotia galloti	0	120	117.65	F	A	620	CUNI	C
		Gallotia galloti	0	122	100.00	M	A (>10 y)	647	CUNI	C
		Gallotia stehlini	1	227.2	65.86	M	A	277	CUNI	C
		Gallotia stehlini	1	150	43.48	M	A	622	CUNI	C
		Gallotia stehlini	1	177	51.30	F	A	116	CUNI	C
		Gallotia stehlini	1	159	46.09	F	A	117	CUNI	C
		Gallotia stehlini	1	144	41.74	F	A	118	CUNI	C
		Gallotia stehlini	1	195	56.52	M	A	119	CUNI	C
		Lacerta agilis	0	91	79.82	M	A	49	CUNI	W
		Lacerta agilis	1	91	79.82	M	A	631	CUNI	W
		Lacerta trilineata major	0	123	80.39	M	A (>4 y)	640	CUNI	C
		Latastia longicauda	0	87.86	79.87	M	A	196	CUNI	W
		Podarcis erhardii	0	67	85.90	M	A	548	CUNI	W
		Podarcis erhardii	1	54	69.23	M	A	549	CUNI	W
		Podarcis melisellensis	0	64.51	96.25	F	A	434	CUNI	W
		Takydromus sexlineatus	0	58.6	96.07	M	A	205	CUNI	W
		Takydromus sexlineatus	0	60.8	99.67	M	A	302	CUNI	W
		Takydromus sexlineatus	0	55	91.67	F	A	372	CUNI	C
		Timon lepidus	1*	150	69.12	M	A	449	CUNI	C
		Timon lepidus	1	135	67.50	F	A	450	CUNI	C
		Timon pater	0	125	92.70	M	A	626	CUNI	C
		Timon tangitanus	1	128	77.11	M	A	43	CUNI	C
		Timon tangitanus	0	150	90.36	M	A	101	Prague Z.	C
		Timon tangitanus	0	152	93.08	F	A	102	Prague Z.	C
		Timon tangitanus	1	123	75.32	F	A	120	CUNI	C
	Teiidae	*Ameiva ameiva*	1	148.9	75.58	M	A	310	CUNI	W
		Aspidoscelis deppei	1	68	73.12	M	A	300	CUNI	W
		Aspidoscelis tigris	0	88.6	89.49	M	A	189	CUNI	W
		Callopistes flavipunctatus	1	245	81.67	F	A	131	CUNI	C
		Salvator rufescens	1	125	20.36	M	SA (0.5 y)	96	Prague Z.	C
		Salvator merianae	1	302	60.28	M	SA	617	CUNI	W
		Tupinambis teguixin	1	297	86.09	M	A	152	NMP	W
Scincoidea	Cordylidae	*Cordylus tropidosternum*	0	88	96.70	M	A	109	Prague Z.	C

Clade	Family	Species	GPC	SVL	SVL_{rel}	Sex	Age	ID	Source	W/C
		Cordylus tropidosternum	0	94	104.33	F	A	110	Prague Z.	C
		Cordylus tropidosternum	0	98	108.77	F	A	111	Prague Z.	C
		Cordylus tropidosternum	0	85	94.34	F	A	157	CUNI	W
		Cordylus tropidosternum	0	78	86.57	F	A	379	CUNI	C
		Ouroborus cataphractus	0	108	93.59	F	A	50	Dubeč Z.	C
		Ouroborus cataphractus	0	107	92.72	F	A	51	Dubeč Z.	C
		Ouroborus cataphractus	0	103	89.25	F	A	52	Dubeč Z.	C
		Ouroborus cataphractus	0	101	81.52	M	A	103	Prague Z.	C
	Gerrhosauridae	*Broadleysaurus major*	0	217	88.57	F	A	89	Prague Z.	C
		Broadleysaurus major	0	216	88.16	F	A	156	CUNI	W
		Broadleysaurus major	0	196	81.67	M	A	164	CUNI	W
		Broadleysaurus major	0	195	81.25	M	A	391	CUNI	C
		Gerrhosaurus flavigularis	0	120	83.33	M	A	97	Prague Z.	C
		Gerrhosaurus flavigularis	0	132	92.96	F	A (6 y)	98	Prague Z.	C
		Gerrhosaurus flavigularis	0	133	93.66	F	A (>9 y)	99	Prague Z.	C
		Gerrhosaurus flavigularis	0	125	88.03	F	A	100	Prague Z.	C
		Gerrhosaurus flavigularis	1	135	93.75	M	A	242	CUNI	C
		Gerrhosaurus flavigularis	1	137.02	95.15	M	A	383	CUNI	W
		Gerrhosaurus nigrolineatus	1	214	87.00	M	A	155	CUNI	W
		Matobosaurus validus	0	225	87.21	F	A	90	Prague Z.	C
		Zonosaurus karsteni	1	125.5	94.36	M	A	299	CUNI	W
		Zonosaurus karsteni	1	135	101.50	F	A	417	CUNI	C
		Zonosaurus karsteni	0	120	90.23	M	A	418	CUNI	C
		Zonosaurus laticaudatus	0	156	115.56	F	A	154	CUNI	W
		Zonosaurus quadrilineatus	0	155	93.94	F	A	410	CUNI	C
		Zonosaurus quadrilineatus	0	145	87.88	M	A	414	CUNI	C
		Zonosaurus quadrilineatus	0	159	96.36	F	A	416	CUNI	C
		Zonosaurus quadrilineatus	0	160	96.97	F	A	632	CUNI	C
	Scincidae	*Bellatorias frerei*	0	208	115.56	F	A	280	CUNI	W
		Bellatorias frerei	1	160	88.89	-	A	629	CUNI	C
		Dasia olivacea	0	107.42	93.41	M	A	191	CUNI	W
		Emoia cyanura	0	48.81	94.85	M	A	169	CUNI	W
		Eumeces schneideri	1	105	79.96	-	A	534	CUNI	C
		Eutropis multifasciata	0	101.4	86.67	M	A	212	CUNI	W
		Chalcides bedriagai	1	76.12	85.53	F	A	313	CUNI	W
		Chalcides ocellatus	0	110.67	82.59	F	A	304	CUNI	W
		Lepidothyris fernandi	1	90	54.55	M	SA	54	Dubeč Z.	C

**Clade**	**Family**	**Species**	**GPC**	**SVL**	**SVL_{rel}**	**Sex**	**Age**	**ID**	**Source**	**W/C**
		Lepidothyris fernandi	1	124	75.15	M	A	55	Dubeč Z.	C
		Lepidothyris fernandi	1	107	84.71	M	A	56	Dubeč Z.	C
		Mochlus sundevallii	0	79.3	90.11	M	A	188	CUNI	W
		Scincus scincus	1	97.68	82.08	M	A	436	CUNI	W
		Scincus scincus	1	94	78.99	F	A	546	CUNI	C
		Tiliqua gigas	0	236	80.00	F	A	163	CUNI	C
		Tiliqua gigas	1	254	88.19	M	A	440	CUNI	W
		Tiliqua gigas	0	340	115.25	F	A	633	CUNI	C
		Tiliqua scincoides	0	274	81.79	F	A	438	CUNI	W
		Tiliqua scincoides	0	287	85.67	F	A	439	CUNI	W
		Trachylepis perrotetii	1	149	82.78	M	A	139	CUNI	W
		Trachylepis quinquetaeniata	1	80	72.07	F	A	147	CUNI	W
		Trachylepis striata	0	86	89.58	M	A	545	CUNI	C
		Tribolonotus gracilis	0	104.52	101.48	M	A	294	CUNI	W
		Tribolonotus novaeguine	0	94.19	91.45	M	A	295	CUNI	W

Histological examinations

Transverse cross-sections through mid-diaphysis of the femur stained with Ehrlich's haematoxylin were examined in four individuals with fully resorbed GPCs and known age to assess whether arrest of longitudinal bone growth is associated with arrest of bone growth in girth. In three out of four femoral bones examined, the lines of arrested growth (LAGs) were not clearly visible (Figure S4 a–c), which might reflect aseasonal growth in captive bred individuals. Yet, tightly spaced rings of laminar bone depositions were observed in the outer cortex of these bones (Figure S4 a–c), a clear indication of decelerated or ceased periosteal growth. In one bone only, we were able to observe LAGs forming the external fundamental system (EFS) (Figure S4 d). Because we know that this individual was at least 7 years old, it seems that this individual has stopped growing in the fourth or fifth year of life. Thus, complete resorption of the femoral GPC is coupled with well-developed EFS in this animal. Taken together, histological examinations performed in this study strongly suggest that periosteal growth is decelerated, if not arrested, in animals with arrested longitudinal growth.

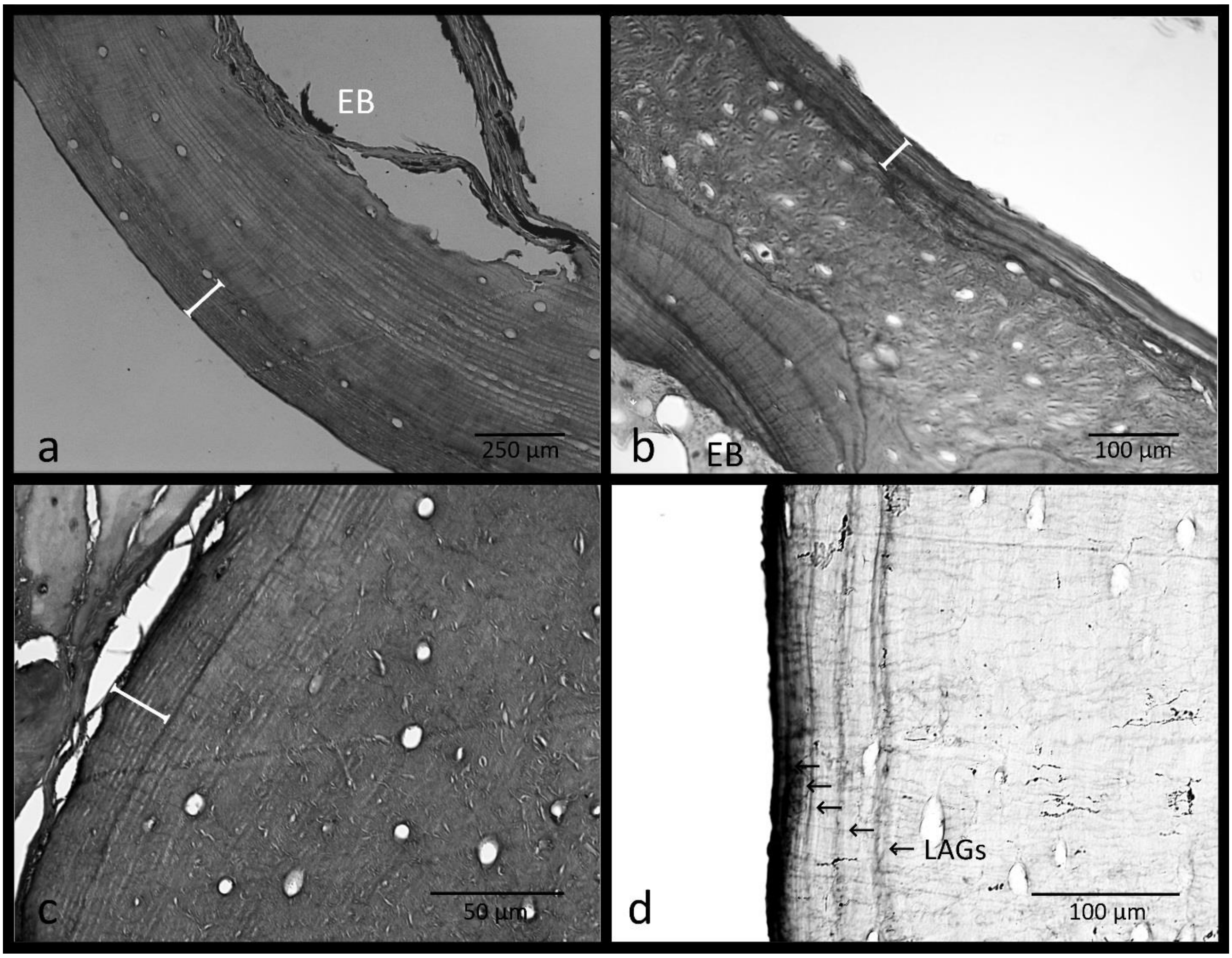

Figure S4. Mid-diaphyseal transverse cross-sections of the femur stained with Ehrlich's Hematoxylin. (a) At least 9-year old female of Yellow-throated plated lizard *Gerrhosaurus flavigularis* (ID 99); (b) At least 5-year old male of Balkan green lizard *Lacerta trilineata major* (ID 640); (c) Nearly 12-year old male of Kuhl's flying gecko *Ptychozoon kuhli* (ID 358); (d) At least 7-year old female of Common leopard gecko *Eublepharis macularius* (ID 187). All these individuals were captive bred and featured complete resorption of the GPC. Note that LAGs are clearly visible in (d) but not in (a–c). White bar in (a–c) marks tightly spaced rings of laminar bone depositions in the outer bone cortex. Arrows in (d) point to LAGs. Abbreviations: EB: endosteal bone, LAGs: lines of arrested growth.

3. Supplementary Discussion

Technical considerations

There are several issues that need to be considered for the sake of an unbiased interpretation of the data presented here. First and foremost, we use the disappearance of the femoral GPC as an indicator of whole-body longitudinal growth cessation, assuming that growth of the femur is synchronized with growth of the axial skeleton. While the timing of GPC degradation is not necessarily synchronous in different bones or the two epiphyses of a single bone [13, 14], this assumption still seems reasonable. Length of the femur is highly correlated (r^2~0.93) with snout-vent length (SVL) in lizards [15], which is in line with a tight coupling and synchronization of growth between the axial and appendicular skeleton.

Second, animals included in our analyses are assumed to be skeletally mature but not senescent. To select adult animals, we preferentially examined individuals that achieved at least 80% of the maximum body length reported in the literature, since 75% is the mean relative size at maturity found in lizards [16]. We were able to collect many fully or nearly fully-grown animals with SVL_{rel} > 80%, with some analysed individuals exceeding the largest size ever reported for a given sex-species category (Table S2). In case of captive bred animals, we confirmed the approximate age and reproductive history of each investigated individual with the breeders. While extensive use of captive bred animals (they constituted two-thirds of the examined specimens) enabled us to exclude immature individuals, growth curves are likely different in wild populations, with captive animals exhibiting faster growth rates and reaching maturity sooner due to controlled environmental and dietary conditions (e.g., [17-19]). Whether this affects the timing of GPC degradation is unknown, but it should not change the inherent growth type. Indeed, our examination of wild and captive bred individuals yielded consistent results.

Finally, it has to be noted that the presence of the GPC in adults cannot be taken as conclusive evidence of indeterminate growth. While the presence of GPCs indicates the potential for longitudinal growth, it does not necessarily mean that the animal is actually growing. Because GPC degradation is triggered by exhaustion of the proliferative potential of growth plate chondrocytes, it has been suggested that complete GPC degradation might not precede, but rather follow the cessation of body growth [20, 21]. Hence, our analysis may overestimate the number of species actually exhibiting extended (potentially indeterminate) adult growth, as we cannot differentiate cases where growth permanently stops while the GPC is still at least partly preserved. Nevertheless, a synchronous timing of GPC degradation, EFS development and body growth arrest has been recently demonstrated in a mammal [22]. Similar complex studies are needed to elucidate the timing and relationship between growth arrest and GPC degradation in squamate reptiles.

Supplementary References:

1. Jakubek J, Jakubek M, Platkevic M, Soukup P, Turecek D, Sykora V, Vavrik D. 2014 Large area pixel detector WIDEPIX with full area sensitivity composed of 100 Timepix assemblies with edgeless sensors. *J. Instrum.* **9**. (doi:10.1088/1748-0221/9/04/c04018)

2. Dudak J, Karch J, Holcova K, Zemlicka J. 2017 X-ray imaging with sub-micron resolution using large-area photon counting detectors Timepix. *J. Instrum.* **12**. (doi:10.1088/1748-0221/12/12/c12024)

3. Frydlova P, Nutilova V, Dudak J, Zemlicka J, Nemec P, Velensky P, Jirasek T, Frynta D. 2017 Patterns of growth in monitor lizards (Varanidae) as revealed by computed tomography of femoral growth plates. *Zoomorphology* **136**, 95–106. (doi:10.1007/s00435-016-0338-3)

4. Frydlova P et al. 2019 Universality of indeterminate growth in lizards rejected: the micro-CT reveals contrasting timing of growth cartilage persistence in iguanas, agamas, and chameleons. *Sci. Rep.* **9**, 14. (doi:10.1038/s41598-019-54573-5)

5. CTVox B. http://bruker-microct.com/products/ctvox.htm

6. Schindelin J, Arganda-Carreras I, Frise E, Kaynig V, Longair M, Pietzsch T, Preibisch S, Rueden C, Saalfeld S, Schmid B, et al. 2012 Fiji: an open-source platform for biological-image analysis. *Nat. Methods* **9**, 676–682. (doi:10.1038/nmeth.2019)

7. Beaulieu JM, O'Meara BC. 2016 Detecting Hidden Diversification Shifts in Models of Trait-Dependent Speciation and Extinction. *Syst. Biol.* **65**, 583–601. (doi:10.1093/sysbio/syw022)

8. Uetz P, Freed P, Hošek J, (eds.). 2019 The Reptile Database

9. Zheng Y, Wiens JJ. 2016 Combining phylogenomic and supermatrix approaches, and a time-calibrated phylogeny for squamate reptiles (lizards and snakes) based on 52 genes and 4162 species. *Mol. Phylogen. Evol.* **94**, 537–547. (doi:10.1016/j.ympev.2015.10.009)

10. Verdenius HHW, Alma L. 1958 A quantitative study of decalcification methods in histology. *J. Clin. Pathol.* **11**, 229-236. (doi:10.1136/jcp.11.3.229)

11. Dawbin WH. 1982 The tuatara Sphenodon punctatus: aspects of life history, growth and longevity. *NZ Wildlife Service, Department of Internal Affairs*

12. Castanet J, Newman DG, Saintgirons H. 1988 Skeletochronilogical data on the growth, age, and population-structure of the Tuatara, Sphenodon punctatus, on Stephens island and Lady-Alice island, New Zealand. *Herpetologica* **44**, 25–37

13. De Buffrenil V, Ineich I, Bohme W. 2005 Comparative data on epiphyseal development in the family Varanidae. *J. Herpetol.* **39**, 328–335. (doi:10.1670/0022-1511(2005)039[0328:cdoedi]2.0.co;2)

14. Tureček A. 2017 The effect of steroid hormones on sexually dimorphic bone growth in geckos. Prague, Charles University

15. Blob RW. 2000 Interspecific scaling of the hindlimb skeleton in lizards, crocodilians, felids and canids: does limb bone shape correlate with limb posture? *J. Zool.* **250**, 507–531. (doi:10.1017/s0952836900004088)

16. Shine R, Charnov EL. 1992 Patterns of survival, growth, and maturation in snakes and lizards. *Am. Nat.* **139**, 1257–1269. (doi:10.1086/285385)

17. Connolly JD, Cree A. 2008 Risks of a late start to captive management for conservation: Phenotypic differences between wild and captive individuals of a viviparous endangered skink (Oligosoma otagense). *Biol. Conserv.* **141**, 1283–1292. (doi:10.1016/j.biocon.2008.02.026)

18. Ritz J, Griebeler EM, Huber R, Clauss M. 2010 Body size development of captive and free-ranging African spurred tortoises (Geochelone sulcata): high plasticity in reptilian growth rates. *Herpetol. J.* **20**, 213–216

19. Ritz J, Clauss M, Streich WJ, Hatt JM. 2012 Variation in Growth and Potentially Associated Health Status in Hermann's and Spur-Thighed Tortoise (Testudo hermanni and Testudo graeca). *Zoo Biol.* **31**, 705–717. (doi:10.1002/zoo.21002)

20. Weise M, De-Levi S, Barnes KM, Gafni RI, Abad V, Baron J. 2001 Effects of estrogen on growth plate senescence and epiphyseal fusion. *P. Natl. Acad. Sci.* **98**, 6871–6876. (doi:10.1073/pnas.121180498)

21. Parfitt AM. 2002 Misconceptions (1): Epiphyseal fusion causes cessation of growth. *Bone* **30**, 337–339. (doi:10.1016/s8756-3282(01)00668-8)

22. Calderon T, DeMiguel D, Arnold W, Stalder G, Kohler M. 2019 Calibration of life history traits with epiphyseal closure, dental eruption and bone histology in captive and wild red deer. *J. Anat.* **235**, 205–216. (doi:10.1111/joa.13016)
